# Cognitive Ecology in Hummingbirds: The Role of Sexual Dimorphism and Its Anatomical Correlates on Memory

**DOI:** 10.1371/journal.pone.0090165

**Published:** 2014-03-05

**Authors:** Paulina L. González-Gómez, Natalia Madrid-Lopez, Juan E. Salazar, Rodrigo Suárez, Pablo Razeto-Barry, Jorge Mpodozis, Francisco Bozinovic, Rodrigo A. Vásquez

**Affiliations:** 1 Instituto de Ecología y Biodiversidad, Facultad de Ciencias, Universidad de Chile, Santiago, Chile; 2 Instituto de Filosofía y Ciencias de la Complejidad, Santiago, Chile; 3 Departamento de Biología, Facultad de Ciencias, Universidad de Chile, Santiago, Chile; 4 Departamento de Ecología, MIII & LINCGlobal, Centro de Estudios Avanzados en Ecología & Biodiversidad, Pontificia Universidad Católica de Chile, Santiago, Chile; University of Sussex, United Kingdom

## Abstract

In scatter-hoarding species, several behavioral and neuroanatomical adaptations allow them to store and retrieve thousands of food items per year. Nectarivorous animals face a similar scenario having to remember quality, location and replenishment schedules of several nectar sources. In the green-backed firecrown hummingbird (*Sephanoides sephanoides*), males are territorial and have the ability to accurately keep track of nectar characteristics of their defended food sources. In contrast, females display an opportunistic strategy, performing rapid intrusions into males territories. In response, males behave aggressively during the non-reproductive season. In addition, females have higher energetic demands due to higher thermoregulatory costs and travel times. The natural scenario of this species led us to compared cognitive abilities and hippocampal size between males and females. Males were able to remember nectar location and renewal rates significantly better than females. However, the hippocampal formation was significantly larger in females than males. We discuss these findings in terms of sexually dimorphic use of spatial resources and variable patterns of brain dimorphisms in birds.

## Introduction

Cognitive abilities, such as learning and memory are pivotal to behavioral performance of animals. These include essential activities such as learning and performing mating displays [1], predator avoidance [2], and food searching [3], among other traits closely linked to fitness [4]. In particular, spatiotemporal memory, which allows individuals to recall time and location of items simultaneously, can be especially important for animals that rely in non-visual cues to retrieve food items. For example, scatter-hoarding species store food in multiple locations dispersed throughout their home range. These animals, like corvids (nutcrackers, jays) and parids (tits and chickadees), can store as much as 100,000 to 500,000 individual caches per year [5], [6], [7]. In this context, several studies have shown that spatial memory abilities are involved in cache retrieval in food-caching species [8], [9], [10], and these species show better cognitive abilities in comparison with non-caching species [11], [12]. The neural mechanisms involved in spatial memory required to retrieve thousands of food items include regions of the medial pallium, such as the hippocampus in mammals [13] or its avian homologue, the hippocampal formation (HF) [14]. In fact, several studies have shown that lesioning the HF in scatter-hoarding bird species severely disrupt food retrieval performance [15], [16].

The adaptive specialization hypothesis (ASH) posits that natural selection may change behavior and its underlying neural mechanisms if such modifications enhance fitness [17], [18], [19]. In the context of spatial memory and its mechanisms at the hippocampus, there are several bodies of evidence supporting ASH. For example, food-hoarding related species have a larger HF than non-hoarding groups [17], [18]. At intraspecific level several findings reveal that populations where caching behavior is observed more often tend to have larger HF than populations that depend less on hoarded food [20], [21]. In addition, several studies have shown a link between sex differences in hippocampal size and differences in the use of spatial information in breeding contexts in birds [22], mammals [23] and fish [24]. Sexually dimorphic neural phenotypes have been observed to occur in species where both sexes have strategies involving different use of space or memory demands. For example, in golden-collared manakins (*Manacus vitellinus*), males perform complex spatial courtship displays and exhibit larger hippocampus and areas related with motor display, while females have a larger ventrolateral mesopallium, which possibly facilitates visual processing in selecting male display traits [25].

Nectarivorous vertebrates, such as hummingbirds, experience a scenario comparable to scattered-hoarding species in which the assessment of nectar quality of individual flowers widely distributed over their home range cannot occur by visual inspection alone, but only after exploitation [26]. Thus, since nectar-rich flowers vary in their concentration, renewal rate and spatial location [27], [28], the ability of hummingbirds to remember where and when nectar-rich flowers will be available results in higher energy rewards than in subjects with poorer memory abilities [29], [3]. Recently, we showed that free-living male hummingbirds are able to remember when (*i.e.*, time) and where (*i.e.*, location) the nectar would be available and are able to match their visits to nectar availability [30]. In several hummingbird species the HF size relative to telencephalic volume has been described two to five times larger than the HF other avian species, even if they are caching songbirds [31] which is consistent with hummingbirds cognitive performance. However, whether memory abilities or HF vary between sexes in hummingbirds is currently unknown. Most hummingbird species (Trochilidae) show morphological and behavioral sexual dimorphisms, mainly related to differences in foraging ecology and resource exploitation strategies [32]. In the green-backed firecrown *(Sephanoides sephaniodes*) males are larger than females and actively defend feeding territories. Females, in turn, are opportunistic [33] and exploit flowers scattered throughout several patches performing rapid intrusions into male territories, as they are aggressively chased away by territorial males during the non-reproductive season [34]. Moreover, females have higher energetic expenditure due to their smaller body size - which implies higher thermoregulatory costs- and longer travel times among several male's territories. Not surprisingly, in this species each sex has differential morphological and physiological traits that improve the energy intake of each strategy. Females show larger wing and bill size and higher intestinal enzymatic activity than males, allowing them to travel longer distances saving energy and have shorter inter-meal times, having the opportunity to feed whenever possible [33, *unpublished data*). Males in contrast, have shorter wings and accurate memory abilities allowing them to effectively defend their territories and keep track of individual flowers exploiting their nectar sources efficiently [33], [3]. The natural scenario of this species led us to compare cognitive abilities and HF in males and females of *S. sephaniodes*. If the territorial condition in males acted as a selective force shaping memory abilities and neuroanatomical linked structures, we predict better cognitive abilities and higher HF volume in males than females of this species.

## Methods

### Ethics Statement

All experimental procedures were conducted according to Chilean laws, legal permits and were approved by the Faculty of Sciences Ethics Committee (Comité de Ética de la Facultad de Ciencias), following the NIH-US guide for the care and use of laboratory animals (NIH publication No. 80-23, 1996). The number of individuals used in this study was minimized to achieve statistical significance and all efforts were made to ameliorate animal discomfort. In particular, animals were euthanized with an overdose of anaesthesia (please see methods). "

### Species and study site

The study was carried out between June and August 2008 and 2010 in a field station located in the Andean foothills within the Estación de Investigaciones Ecológicas Mediterráneas of the Pontificia Universidad Católica de Chile, San Carlos de Apoquindo, central Chile (33°23′S, 70°31′W, 1100 m above sea level). In the behavioral tests subjects were 6 males (6.83 ± 0.2 g body weight, mean ± se) and 9 females (5.42±0.17 g) *S. sephaniodes*, captured with mist nets and released at the end of the experiment. Three females did not complete the behavioral protocol and were released after temporal training (see below). Specimens for brain analysis (4 females and 3 males) were captured with mist nets at the same field station.

### Behavioral experimental protocol: Testing elements of What, Where and When

The methodology used to test the ability to remember what, where and when the best nectar sources would be available was previously described in detail in González-Gómez et al. 2011b [30]. However, this experiment was performed in captivity since territorial males actively chase females. The experiment was carried out in a 6×6×5 m aviary exposed to the field, and comprised a habituation period of 15 h, in which the subjects got used to the feeders. Subjects were tested individually in the aviary. For the habituation period, we provided nectar *ad libitum* in two feeders filled with 100 ml of 25% sucrose (w/w in water) each. Feeders were located 1.5 m above the ground, attached to the aviary structure, and consisted of a 100 ml glass water dispenser for rodents wrapped with red paper.

**Figure 1:**
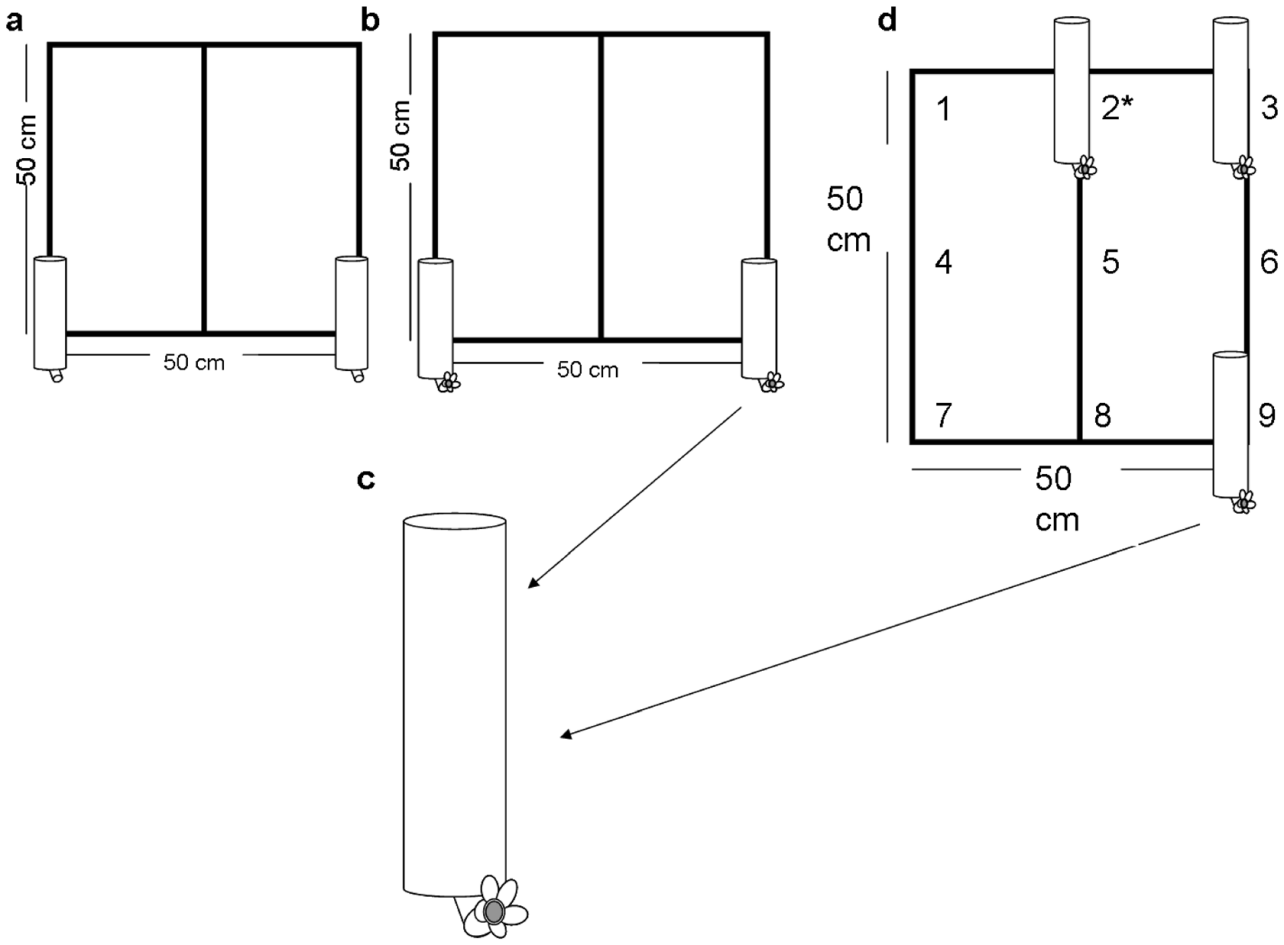
Experimental grid used in the nectar quality training. (a) Each feeder contents 100(b) Experimental grid used in temporal training. Two artificial flowers -mounted on empty feeders- with different nectar qualities and different nectar renewal rates were attached to the experimental grid. (c) Enlarged image of artificial flowers used in the temporal training and in the test of elements of what, where and when memory. (d) Experimental grid to test the ability to recall elements of what, where and when the best nectar was available. Numbers indicate nine flower locations. Only three positions (i.e. three flowers mounted on empty feeders) were used in each experiment [three flowers are shown as an example]. *High quality artificial flower.

As previously described in González-Gómez et al. 2011b [30], the protocol has two training periods:

- *Nectar quality training*. To show the subject that there were two different nectar qualities available, we replaced the training feeders with two feeders (Fig. 1a), one of them with 15% sucrose (low quality) and the other one with 30% sucrose (high quality). The arrangement of the feeders was randomly selected and it was maintained until the subject had visited both feeders at least 3 times each.
- *Temporal training*. To present subjects with nectar renewal rates associated with nectar quality, following the nectar quality training, we replaced the previously used grid with an identical vertical grid but with an artificial red flower at the base of each feeder (Fig. 1b) that hold a quantity small enough to be consumed by a green-backed firecrown in just one visit. Each artificial flower contained 60 µL of nectar and it was made of an orange syringe needle cap with red paper petals mounted horizontally in an empty training feeder (Fig. 1c). The high quality flower (i.e., 30% sucrose) was refilled every 10 min after it was drained. The low quality flower (i.e., 15% sucrose) was refilled every 5 min. In order to prevent hummingbirds from using the filling bouts as a visual cue of nectar renewal rate, we randomly performed 5 sham fillings per hour where the observer approached the grid and mimicked a filling bout but delivering no nectar in the artificial flowers. The schedule of the sham fillings was inconsistent with the nectar renewal rate assigned to nectar qualities. The temporal training was maintained for one hour or until three consecutive visits occurred within a nectar refill interval (see below).

### Testing Elements of What, Where and When

This experiment was designed to test the ability of hummingbirds to remember the position and renewal rate of high and low rewarding nectar sources. In order to match their visits to the rewarding flowers, individuals had to apply the information acquired during the training periods, relating a particular nectar concentration with its nectar renewal rate. Following the training periods we presented the hummingbirds with a new grid with 3 artificial flowers randomly located among 9 possible positions (Fig. 1d). Each artificial flower contained 60 µL of either a 30% sucrose solution (‘high quality’, refilled every 10 min, one flower) or 15% sucrose solution (‘low quality’, refilled every 5 min, two flowers). Each trial had two consecutive phases: ‘search’ and ‘return’. In the ‘search’ phase the hummingbird was free to visit the array until it fed from the most rewarding flower and left the grid. The second phase (i.e., ‘return’ phase) started when the hummingbird visited the grid again and ended when the bird left the grid. In the return phase individuals faced the same array of flowers that in the search phase. For each trial we scored the number, position and quality of the visited artificial flowers and the time elapsed between both phases of each trial. After finishing the return phase, the trial was ended. Ten minutes later, new positions for the artificial flowers were randomly selected for the next trial.

### Hippocampal morphometric analysis

We used 3 male and 4 female *S. sephanoides*, captured in the same site of study after all behavioral experiments. They were euthanized with an overdose of anaesthetic (ketamine 75 mg/kg and xylazine 5 mg/kg, i.p.) followed by transcardial perfusion of phosphate-buffered saline (PBS, 0.1 M) and 4% paraformaldehyde in PBS. The brains were carefully dissected and cryoprotected in 30% sucrose. We cut 45 µm coronal sections and mounted series spaced 180 µm for cresyl-violet Nissl staining. The slides were photographed using a bright field microscope (BX 60; Olympus Optical) coupled to a camera with inbuilt analysis software (Spot Advanced). To measure volumes of both the hippocampal formation (HF) and the telencephalon we used the Cavalieri method of unbiased stereology [31] as implemented in Stereo Investigator (Microbrightfield Inc., Colchester, VT, USA). All slides were coded to avoid experimenter bias. We measured the HF and telencephalon on every third section of the specimens with a 200 mm grid using the same HF borders as earlier studies [14], [15]. The errors coefficients of HF and telencephalon were 0.011 (range: 0.012–0.013) and 0.0068 (range: 0.006–0.018) respectively.

### Statistical analyses

During temporal training, we compared the inter-meal interval among the high- and low-quality feeders and individual performance with repeated-measures ANOVAs. The time intervals of the three last visits to the high-quality feeder and to the low-quality feeder were compared with Student's t-test.

During testing, we compared the average number of feeders visited by each individual, the time elapsed between visits in both phases to both quality flowers and individual performance differences in both phases of the experiments with Repeated-measures ANOVAs [35]. We used Tukey's HSD test as *post hoc* tests for equal sample sizes. We compared difference between the mean number of foraging intervals and nectar availability (5 min for low-quality and 10 min for high-quality) using a 1000 bootstrap sampling to calculate the 95% CI and compare t with each individual's performance. All statistical analyses were performed using JUMP 10.0 (SAS, Inc) and R. Results are presented as means ± SE.

The energetic consequences of the ability to keep track of the best nectar sources was assessed by transforming the mean of nectar obtained to energy units (joules, J), corrected for body mass. To assess the differences between males and females, we performed a nested analysis of variance for balanced samples [35]. Data were tested for autocorrelation and they met the assumptions for each test.

We explained the effect of sex and telencephalon volume on the HF volume with general linear model (GLM, normal distribution, identity function link) [36].

## Results

### Behavioral experimental protocol

During *temporal training*, three females did not match at least 3 of their visits with nectar renewal rates within an hour, therefore we considered they did not learn the temporal training and were released before to complete the test. We captured three additional females to balance the statistical design. Thus, 6 males and 6 females were completely tested (*i.e.*, training and testing). To compare the revisit time among the high- and low-quality feeders, we calculated the time elapsed between the last three visits to low and high-quality flowers. The mean inter-meal interval of the last three visits was significantly longer when subjects were visiting the high quality feeder (10.19±0.25, min ± mean ± *SE*, *N* = 30) than when visiting low-quality feeders (5.37±0.17 min, *N* = 30, repeated measures ANOVA F(1,11) = 234.29, *p*<.001). The time intervals of the three last visits to the high-quality feeder and to the low-quality feeder did not differ significantly from the respective replenishment intervals of the feeders (one-sample t test against 10 min for high-quality feeder: t(11) = 0.87, *p* = 0.40; one-sample t test against 5 min for low quality feeder: *t*(11) = 2.14, *p* = 0.06

### Testing elements of What, Where and When

**Figure 2:**
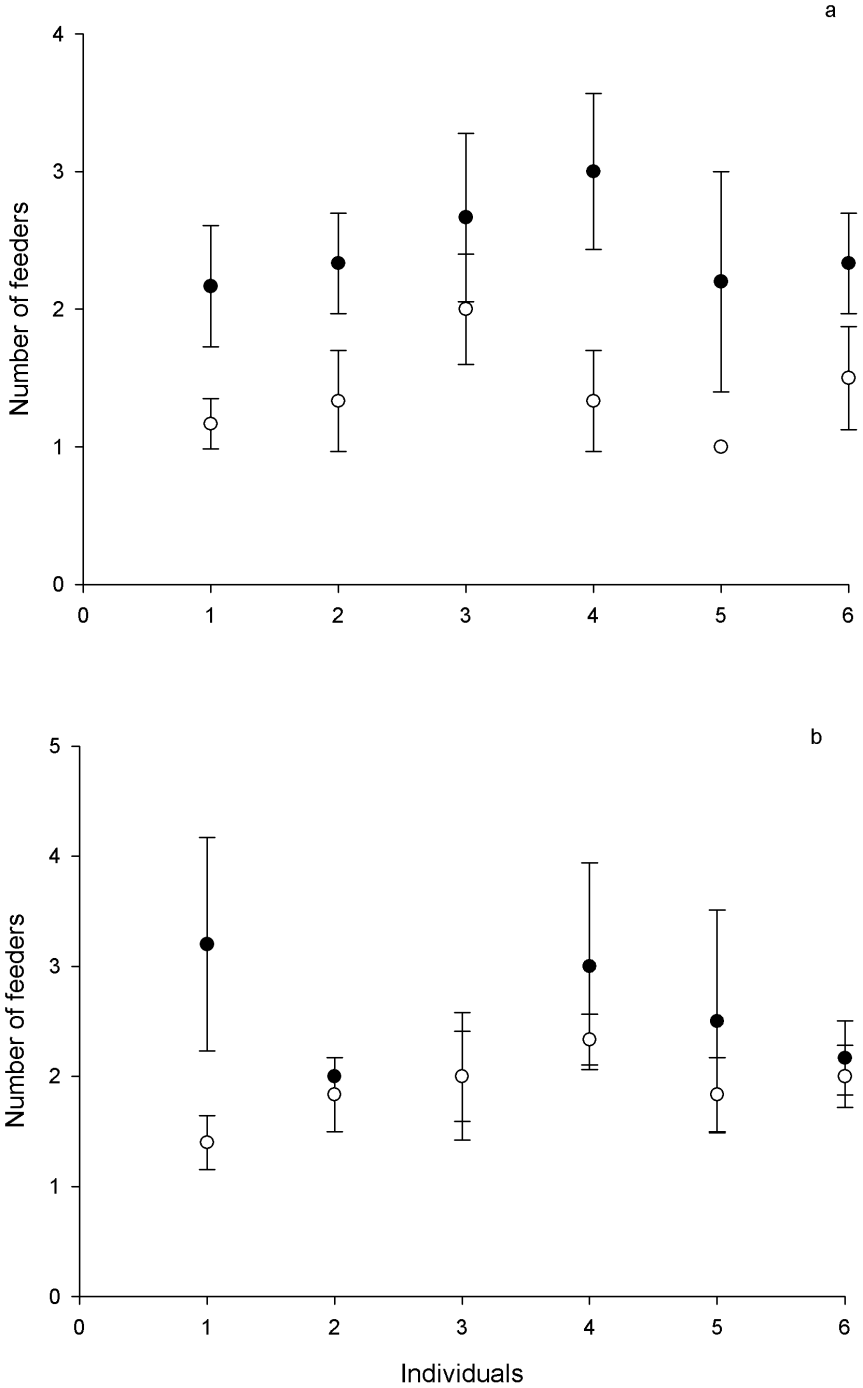
Number of feeders visited in the search phase (black dots) and in the return phase (white dots) of the experiment testing the ability of Green-backed Firecrown hummingbirds to recall what, where and when (*N* = 6 trials). Male's performance in the upper panel, female's performance in the lower panel. Data are presented as mean ± *SE*.

During testing, we aimed to evaluate the ability of each individual to remember the location where the nectar was available by comparing the averages of the number of feeders visited by each individual in both phases of the experiments. If individuals can remember where the best nectar is, then the number of visits in the ‘search’ phase of the experiments will be significantly higher than in the ‘return’ phase. Males visited significantly more feeders in the search phase (2.45±1.14 feeders, mean ± *SE*, *N* = 6 trials; Fig. 2a) than in the return phase (1.38±0.15, repeated measures ANOVA *F*(1,5) = 24.56, *p*<0.001). There was no significant difference among individuals (repeated measures ANOVA *F*(1,36) = 0.80, *p* = 0.55; Fig. 2a). One individual (subject number 3; Fig. 2a) did not find the best nectar location in fewer visits during the return phase than during the search phase.

In contrast, in the female performance there were no significant differences among the number of feeders visited in the ‘search’ phase (2.47±0.23 feeders, mean ± *SE*, *N* = 6 trials; Fig. 2b) than in the ‘return’ phase (1.9±0.14, repeated measures ANOVA *F*(1,5)  = .49, *p* = 0.77). Individually, just one of six females performed fewer numbers of visits in the ‘search’ than in the ‘return’ phase (subject 1; Fig. 2b). We did not find individual differences among females performance (repeated measures ANOVA *F*(1,36) = 3.67, *p* = 0.06; Fig.2b).

**Figure 3:**
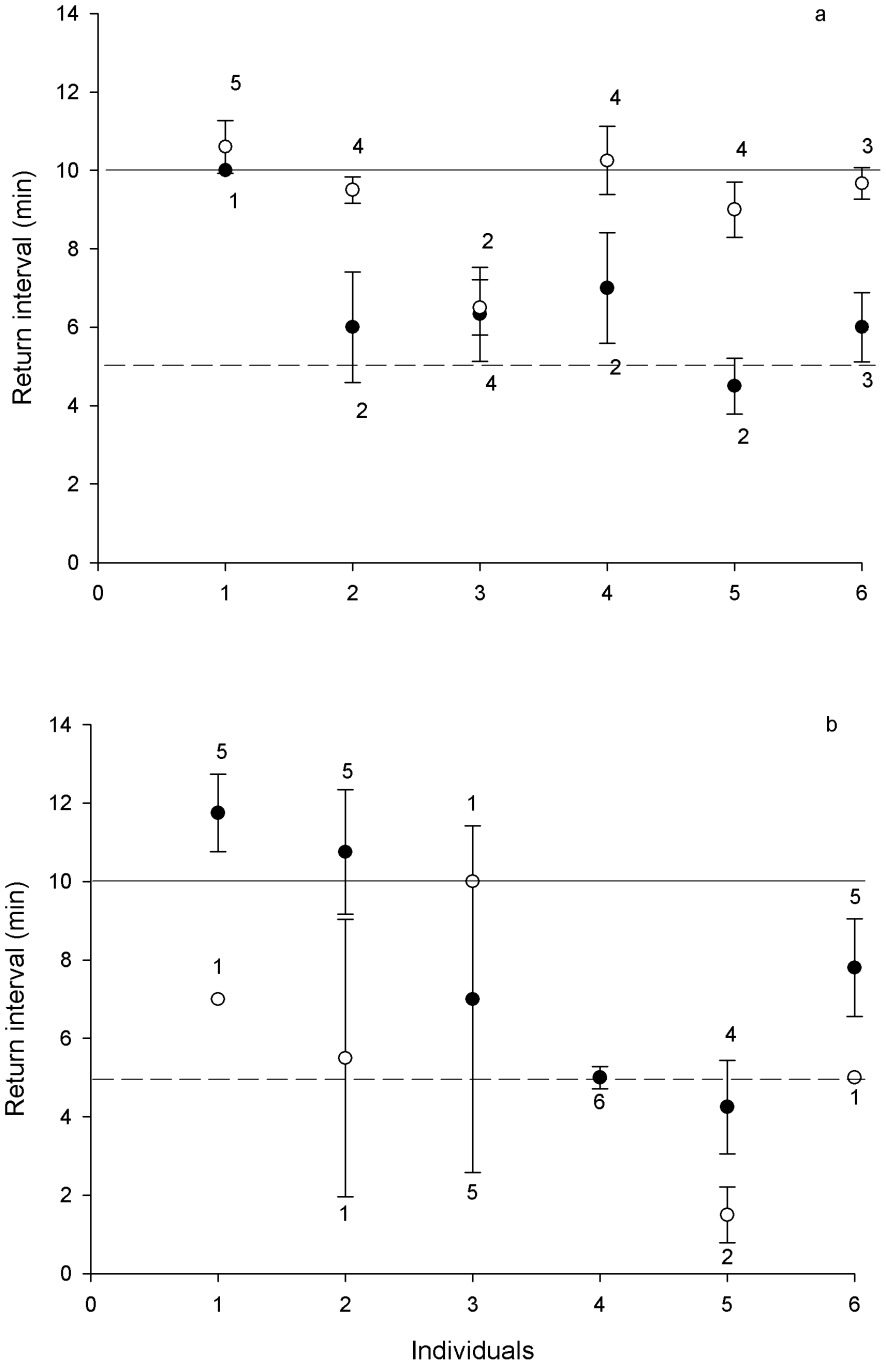
Return intervals of males (upper panel) and females (lower panel) to high quality (white dots) and low-quality (black dots) feeders between the search and return phase of the experiment. Number of visits (mean ± *SE*) to both nectar qualities is shown. Solid line: renewal interval of high-quality nectar feeders; dashed line: renewal interval of low-quality nectar feeders. *Return interval differed significantly from the nectar renewal interval, 1000 bootstrap sampling (High quality nectar, 95% CI: 11.1–9.75 min, Low quality nectar, 95% CI: 6.15, 4.75 min).

In order to assess the individual's ability to remember the nectar renewal rates of each type of flowers (i.e., high and low qualities), we registered the time elapsed between visits in both phases to both quality flowers. In the return phase when males were revisiting high quality nectar flowers they performed significantly longer intervals between visits (9.57±0.32 min) than when they were revisiting ‘low’ quality feeders (5.93±0.52 min; repeated-measures ANOVA, *F*(1,5) = 12.89, *p*<0.01; Fig. 3a). Females showed no significant difference in revisit time to ‘high’ quality feeders as compared to ‘low’ quality feeders (5.1±1.18 min vs. 7.57±0.74 min; Repeated-measures ANOVA, *F*(1,5) = 0.01, *p* = 0.90; Fig. 3b). The energy intake of females was 0.44 times lower than in males, consistent with females being unable to keep track of the location, quality, and nectar availability intervals in the best flowers (nested ANOVA, Table 1). As an example of this marked dimorphism, the male who had the best performance (individual number 6) obtained 11.63 times more energy than the female with the poorest performance (individual number 5), while the male with the worst performance obtained 3.6 times more energy than this female.

**Table 1:** Effect of cognitive ability in energy reward in males and females of *S. sephaniodes*.

Source of variation	SS	Df	MS	F
Sex	6992	1	6992	10.884**
Sex (Individuals)	14588	10	1459	2.271 *
Error	35974	56	642	

Nested ANOVA. *p<0.05, ** p<0.001.

### Hippocampal formation

**Figure 4:**
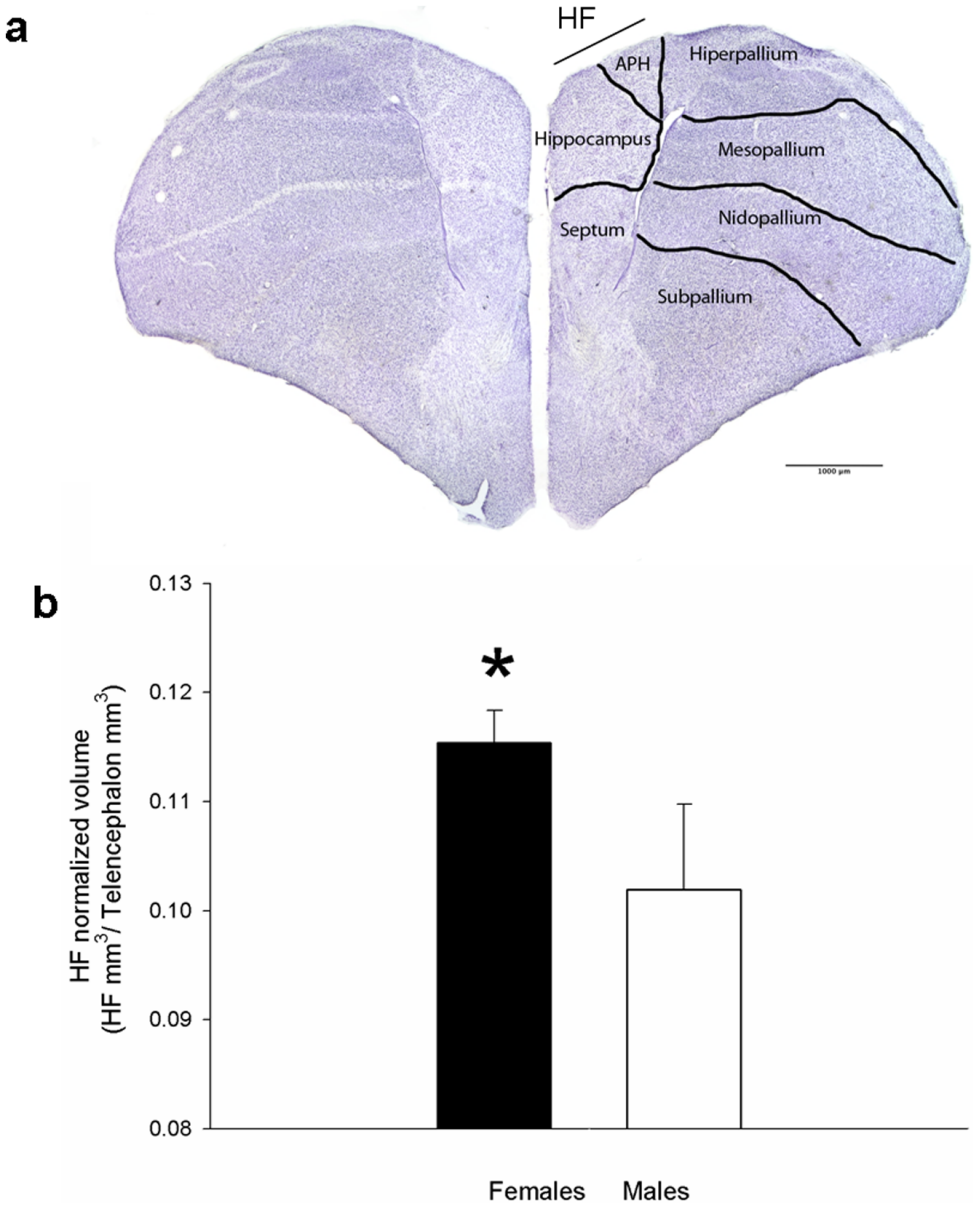
Coronal section through the telencephalon of the Green-backed Firecrown hummingbird showing the Hippocampal formation (i.e., hippocampus and area parahippocampalis (APH)) in the medial-dorsal aspect. Scale bar: 1000 microns (a). (b) The HF is significantly larger in females (13.96±0.81 mm^3^, mean ± se) than males (12.04±0.8) respect to the telencephalon volume (121.31±8.30, 117.81±5.36, respectively). Please note the statistical analysis was performed using telencephalon volume as a covariable [36]. Data are presented as mean ± *SE*. * *p*<0.05.

In *S. sephanoides* the hippocampal formation (HF) corrected by telencephalon volume is 1.13 times larger in females than males (*p* = 0.03; Table 2, Fig. 4).

**Table 2:** Parameter estimates (Bayesian posterior models) for three GLM models of effects of telencephalon volume (mm3), and sex on the size of Hp, APH and HF.

Whole model	Term	Parameter estimate	Chi-Square	-LogLikelihood
HF (Hp+APH)			10.71**	8.21
	Telencephalon volume	0.112	8.02**	
	Sex	0.76	4.53*	

* *p*<.05, ** *p*<.001.

## Discussion

Male hummingbirds showed the ability to learn where and when nectar sources were available and match their foraging visits accordingly. Males visited smaller number of flowers in the second phases of the experiments and were able to match their visits to different nectar schedules. Thus, when individuals were visiting high quality nectar sources, they performed longer inter-visit periods than when visiting poor quality nectar sources. In roughly a third of the trials individuals reached the high quality feeder in the first visit, therefore in the second phase they had just to “return to same” feeder. It is remarkable that hummingbirds were able to change that rule in successive trials depending on what they experienced in the first phase of the experiment. Our results confirm our previous findings that male hummingbirds can remember what, where and when nectar will be available [30], [37]. In contrast, females were unable to recall the location or nectar renewal schedules of nectar sources regardless of their quality.

Several species of birds [38], [39], [40] and mammals [41], [42], [43] show sexual differences in memory abilities, which could be a response to selective forces acting differentially on each sex [4], [44]. In the context of ASH, our results suggest that in males of the green-backed firecrown, spatial and temporal memory abilities could have co-evolved in response to their territorial behavior and regular feeding events, based on remembering the exact location, quality, and time availability of potential resources. In turn, the increase in gained energy could stimulate the territorial defense. Females, in contrast, have a strong selection on morphological and physiological traits that make them able to forage as intruders within male territories [33]. The opportunistic strategy observed in females appears to be consistent with their poor cognitive performance at the micro-scale, with low incentives to remember individual flower characteristics in a focal territory. Instead, females probably can remember features of several patches at a larger spatial scale, such as overall flower abundance or male aggressiveness.

We found HF volume in *S. sephaniodes* to be consistent with previous values reported by Ward et al. 2012. However, female's HF volumes corrected by the 3/4 power of body mass [45] are more similar to *Selasphorus rufus* volumes than to values of males *S. sephaniodes*. Interestingly, both species perform long distance migrations, a highly demanding process in terms of spatial memory and present higher HF volumes than year-round resident species which is consistent with previous studies in passerine birds [46]. Our finding of intra-specific differences in hippocampus size has been reported linked to seasonal changes [47] and/or reproductive behavior. For example, in cowbirds which are nest-parasitic species, the HF is larger in females than in males only in those species in which the females search for host-nests alone [48], [22]. In addition, these differences seem to be seasonal and are found during the breeding season but not in the non-breeding season [49].

Our result of larger HF volume in females than males may seem to contradict with their behavioral performance. However, there are several potential explanations for these results. First, as we mentioned before females may have similar or even better cognitive abilities than males at a larger spatial scale. Given that females do not defend territories as males do [33], they may have larger home ranges, so they keep track of many different food sources for which a larger hippocampus would be adaptive. It is possible that the scale of our experimental setup was not able to detect these abilities. In addition, since males are aggressive toward male and female intruders during the non-reproductive season [33], the ability of females to remember the location of available territories or those occupied by less aggressive males could allow them to maximize the exploitation of individual patches. Lastly, although volumetric measurements alone might not be the best proxy for neural changes in brain structures linked to changes in behavior, they might still reflect morphological specializations [50]. Our very preliminary survey on cell density suggest that sex differences in this species are not related with differences in the number of neurons in the HF which would imply higher mean distance between neuronal cell bodies and differences in dendritic arborization of the neurons or different number of glial cells [51]. Undoubtedly, exhaustive steorological studies are required to confirm these ideas.

Certainly, the generality of our findings needs to be tested at larger spatial scales and in other species of diurnal animals to determine whether our conclusions apply to other systems. For instance, the properties of different habitats sizes and territoriality could be further measured in conjunction with new food manipulation experiments
