# Correction: Cognitive Ecology in Hummingbirds: The Role of Sexual Dimorphism and Its Anatomical Correlates on Memory

**DOI:** 10.1371/journal.pone.0099750

**Published:** 2014-06-02

**Authors:** The PLOS ONE Staff

One of affiliations for author Pablo Razeto-Barry is missing from the published article. Dr. Razeto-Barry is affiliated with the Universidad Diego Portales, Vicerrectoría Académica, Manuel Rodríguez Sur 415, Santiago, Chile, as well as the Instituto de Filosofía y Ciencias de la Complejidad, Santiago, Chile.
