# Correction: Cognitive Ecology in Hummingbirds: The Role of Sexual Dimorphism and Its Anatomical Correlates on Memory

**DOI:** 10.1371/journal.pone.0114671

**Published:** 2014-11-25

**Authors:** The PLOS ONE Staff

There is an error in the funding statement. Please refer to the correct funding statement below.

This work received funding from CONICYT (PGG and RS), FONDECYT 1130015 (FB) and FONDECYT N°* 3120078 (PRB),* FONDECYT 1090794, 1060186; ICM-P05-002; and PFB-23-CONICYT (RAV). The funders had no role in study design, data collection and analysis, decision to publish, or preparation of the manuscript.
